# Host metabolomic responses in recurrent *P. vivax* malaria

**DOI:** 10.1038/s41598-024-54231-5

**Published:** 2024-03-27

**Authors:** Michael N. Yakubu, Victor I. Mwangi, Rebeca L. A. Netto, Maria G. C. Alecrim, Jessica R. S. Alves, Anne C. G. Almeida, Gabriel F. Santos, Gesiane S. Lima, Lucas S. Machado, Hector H. F. Koolen, Tiago P. Guimarães, Andrea R. Chaves, Boniek G. Vaz, Wuelton M. Monteiro, Fabio T. M. Costa, Marcus V. G. Lacerda, Luiz G. Gardinassi, Gisely C. de Melo

**Affiliations:** 1 State University of Amazonas (UEA), Manaus, Amazonas, Brazil/Tropical Medicine Foundation-Dr. Heitor Vieira Dourado (FMT-HVD), Manaus, Amazonas Brazil; 2 Institute of Biology, Laboratory of Tropical Diseases-Prof. Dr. Luiz Jacintho da Silva (LDT-LJS), Department of Genetics, Evolution, Microbiology and Immunology, State University of Campinas (UNICAMP), Campinas, São Paulo Brazil; 3 Institute of Tropical Pathology and Public Health, Federal University of Goiás, Goiânia, Goiás Brazil; 4 Chromatography and Mass Spectrometry Laboratory, Institute of Chemistry, Federal University of Goiás, Goiânia, Goiás Brazil; 5 Department of Microbiology, Federal University Wukari, Wukari, Taraba State Nigeria; 6 Postgraduate Program in Sciences Applied to Hematology, PPGHUEA/HEMOAM), Manaus, Amazonas Brazil; 7 Leônidas & Maria Deane Institute/Oswaldo Cruz Fundation (ILMD/Fiocruz Amazônia), Manaus, Brazil; 8 Metabolomics and Mass Spectrometry Research Group, State University of Amazonas, Manaus, Amazonas Brazil; 9 University of São Paulo, Ribeirão Preto School of Nursing, Department of Maternal and Child Nursing and Public Health, Ribeirão Preto, Brazil

**Keywords:** Pathogens, Computational biology and bioinformatics

## Abstract

Malaria is the leading parasitic disease worldwide, with *P. vivax* being a major challenge for its control. Several studies have indicated metabolomics as a promising tool for combating the disease. The study evaluated plasma metabolomic profiles of patients with recurrent and non-recurrent *P. vivax* malaria in the Brazilian Amazon. Metabolites extracted from the plasma of *P. vivax*-infected patients were subjected to LC–MS analysis. Untargeted metabolomics was applied to investigate the metabolic profile of the plasma in the two groups. Overall, 51 recurrent and 59 non-recurrent patients were included in the study. Longitudinal metabolomic analysis revealed 52 and 37 significant metabolite features from the recurrent and non-recurrent participants, respectively. Recurrence was associated with disturbances in eicosanoid metabolism. Comparison between groups suggest alterations in vitamin B6 (pyridoxine) metabolism, tyrosine metabolism, 3-oxo-10-octadecatrienoate β-oxidation, and alkaloid biosynthesis II. Integrative network analysis revealed enrichment of other metabolic pathways for the recurrent phenotype, including the butanoate metabolism, aspartate and asparagine metabolism, and N-glycan biosynthesis. The metabolites and metabolic pathways predicted in our study suggest potential biomarkers of recurrence and provide insights into targets for antimalarial development against *P. vivax*.

## Introduction

Malaria is a parasitic disease that remains a major global public health problem. The two *Plasmodium* species posing the greatest threat to humans are *P. falciparum* and *P. vivax*^1,2^. While *P. falciparum* is responsible for global mortality, *P. vivax* is the most widely geographically distributed malaria parasite. Vivax malaria is predominant in the Americas, South-East Asia, Western Pacific, and Eastern Mediterranean regions. In Brazil, 84% of malaria infections are caused by *P. vivax*, predominantly affecting people in the Amazonian region, resulting in high morbidity rates and significant economic impacts^3^.

For a long time, vivax malaria was thought to be a benign infection, however, this rationale has changed recently^4,5^. Considering that *P. vivax* can induce recurrences, this poses a significant obstacle to malaria elimination^6,7^. Recurrence is a newly detectable episode of blood-stage parasitemia occurring after receiving anti-malarial treatment for a previous malaria episode. This recurrence may arise from reinfection, recrudescence, or relapse^8–10^. Reinfection results from a new mosquito bite by a vector. Recrudescence is the reappearance of parasite forms in the bloodstream after they have been treated to undetectable levels for a period of time, with the presence of plasma concentrations of chloroquine (CQ) and desethylchloroquine (DCQ) higher than 100 ng/mL^11–13^. Relapse occurs due to the reactivation of the dormant "hypnozoite" located in the liver^14^. Previous research indicated that relapses account for approximately 90% of *P. vivax* malaria cases in several countries where the disease is prevalent^15^.

Metabolites including lipids, amines, organic acids, and amino acids, reflect an individual’s health or disease state^16,17^. Minor changes in gene expression during a disease can affect the production of downstream metabolites. Due to the relationship between metabolites and cellular systems, detection of certain metabolites can accurately depict the cell's condition and serve as biomarkers of disease diagnosis and prognosis, development of novel drug targets, or vaccine candidates^18^. Therefore, the omics science has the potential to provide insights into better understanding of vivax malaria and contribute to the development of new approaches to the disease elimination. Metabolomics and other multi-omics methods, have progressively gained significance as valuable tools in the field of biomedical research^19^. Metabolomics has the potential to revolutionize malaria research by providing a vital groundwork for new drug and vaccine, bridging the gap between basic research and the treatment of malaria patients. This can occur via the understanding of physiological and pathological processes and discovery of biomarkers of recurrent malaria^5,12,20–26^.

Previous studies in *P. vivax* metabolomics have identified various metabolic pathways including xenobiotics, glycosphingolipids, aspartate and asparagine, purine and pyrimidine, vitamin B6, methionine and cysteine, fatty acid, urea cycle, stearoylcarnitine, phosphocholine, glycerophosphocholine, biliverdin, bilirubin, palmitoylcarnitine, oleic acid and omega-carboxytrinor-leukotriene B4 metabolism^5,12,22,27,28^. Additionally, important biomarkers like plasmodial lactate dehydrogenase, histidine-rich protein II, and urinary ornithine have been identified^22,29,30^. In certain instances, elevated serum levels of these metabolites were suggestive of malaria or pointed to impaired organ function^22^.

Currently, few studies have provided information about disturbances of metabolic pathways in recurrent vivax malaria, creating gaps in our knowledge of the interaction between malaria and its host. This study aimed to determine changes in metabolites and metabolic pathways associated with *P. vivax* recurrence.

## Results

### Socio demographic characteristics, and laboratory data of the patients.

Fifty-one cases (recurrent malaria) and fifty-nine controls (non-recurrent malaria), totaling 110 malaria patients were recruited. Overall, the proportion of males was higher than females (73.3% v/s 26.4%), in both groups. The age distribution was similar between cases and controls, with a median age of 40.5 (IQR: 29.0–50.0). There was also no significant difference in the ethnic distribution of the patients, sexual and asexual parasites density and parasite clearance time between groups (p > 0.05). The distribution of ethnicity was higher in individuals with brown skin (74/110, 67.3%), followed by whites (24/110, 21.8%) and blacks (12/110, 10.9%). Socio-demographic and laboratory data showed no significant differences between the cases or control participants (Table 1).

**Table 1:** Sociodemographic and laboratory data of cases and control participants.

Characteristics	Total	Cases	Controls	p-value
	N = 110	N = 51	N = 59	
Gender (%)				0.850
Male	81/110 (73.6%)	38/51 (74.5%)	43/59 (72.9%)	
Female	29/110 (26.4%)	13/51 (25.5%)	16/59 (27.1%)	
Age, years	40.5 (29.0–50.0)	40.0 (30.0–51.0)	41.0 (29.0–50.0)	0.730
Weight, kg	75.0 (63.0–83.0)	76.0 (63.0–82.9)	73.5 (64.0–83.1)	0.480
BMI, kg/m^2^	26.8 (23.4–30.1)	28.0 (23.1–30.3)	26.4 (23.4–30.0)	0.410
Ethnicity (%)				0.320
Mixed race	74/110 (67.3%)	33/51 (64.7%)	41/59 (69.5%)	
White	24/110 (21.8%)	10/51 (19.6%)	14/59 (23.7%)	
Black	12/110 (10.9%)	8/51 (15.7%)	4/59 (6.8%)	
Parasite density (IQR)				
Asexual D1, parasites/μL	3949.8 (2080.0–7210.2)	3878.4 (2538.9–6346.0)	4134.4 (2064.4–7472.0)	0.850
Asexual D2, parasites/μL	134.4 (38.4–618.8)	100.2 (28.9–394.9)	217.6 (38.4–717.8)	0.300
Asexual D3, parasites/μL	7.4 (0.0–39.2)	9.0 (0.0–30.6)	5.4 (0.0–43.3)	0.960
Asexual DR, parasites/μL		2350.3 (251.9–6282.7)		
Sexual D1, parasites/μL	24.7 (0.0–73.8)	14.1 (0.0–71.8)	32.0 (0.0–80.5)	0.350
Sexual D2, parasites/μL	0.0 (0.0–42.8)	0.0 (0.0–20.1)	13.3 (0.0–61.2)	0.035
Sexual D3, parasites/μL	0.0 (0.0–0.0)	0.0 (0.0–0.0)	0.0 (0.0–0.0)	0.730
Sexual DR, parasites/μL		0.0(0.0–59.5)		
Parasite clearance time, days				
Assexual	4.0 (3.0–5.0)	3.0 (3.0–5.0)	5.0 (3.0–5.0)	0.980
Sexual	3.0 (0.0–3.0)	2.0 (0.0–3.0)	3.0 (2.0–3.0)	0.100
Copy number (copy numbers/μL)				
Asexual, D0	–	1712.9 (322.8–9368.1)*	1172.0 (245.6–4939.5)	0.285
Asexual, DR	–	39.3 (7.7–2096.9)*	–	–
Cases on recurrence days, (%)				
D42		24/51 (47.1)		
D63		8/51 (15.7)		
D90		7/51 (13.7)		
D120		4/51 (7.8)		
D150		5/51 (9.8)		
D180		3/51(5.9)		

*DR* Day of recurrence, *D1, D2, D3…* Day 1, Day 2, Day 3.

*There was a significant difference between the DNA copies of D0 and DR of the recurrent group (p value = 0.0001).

### Differential abundance of metabolite features across time

**Figure 1:**
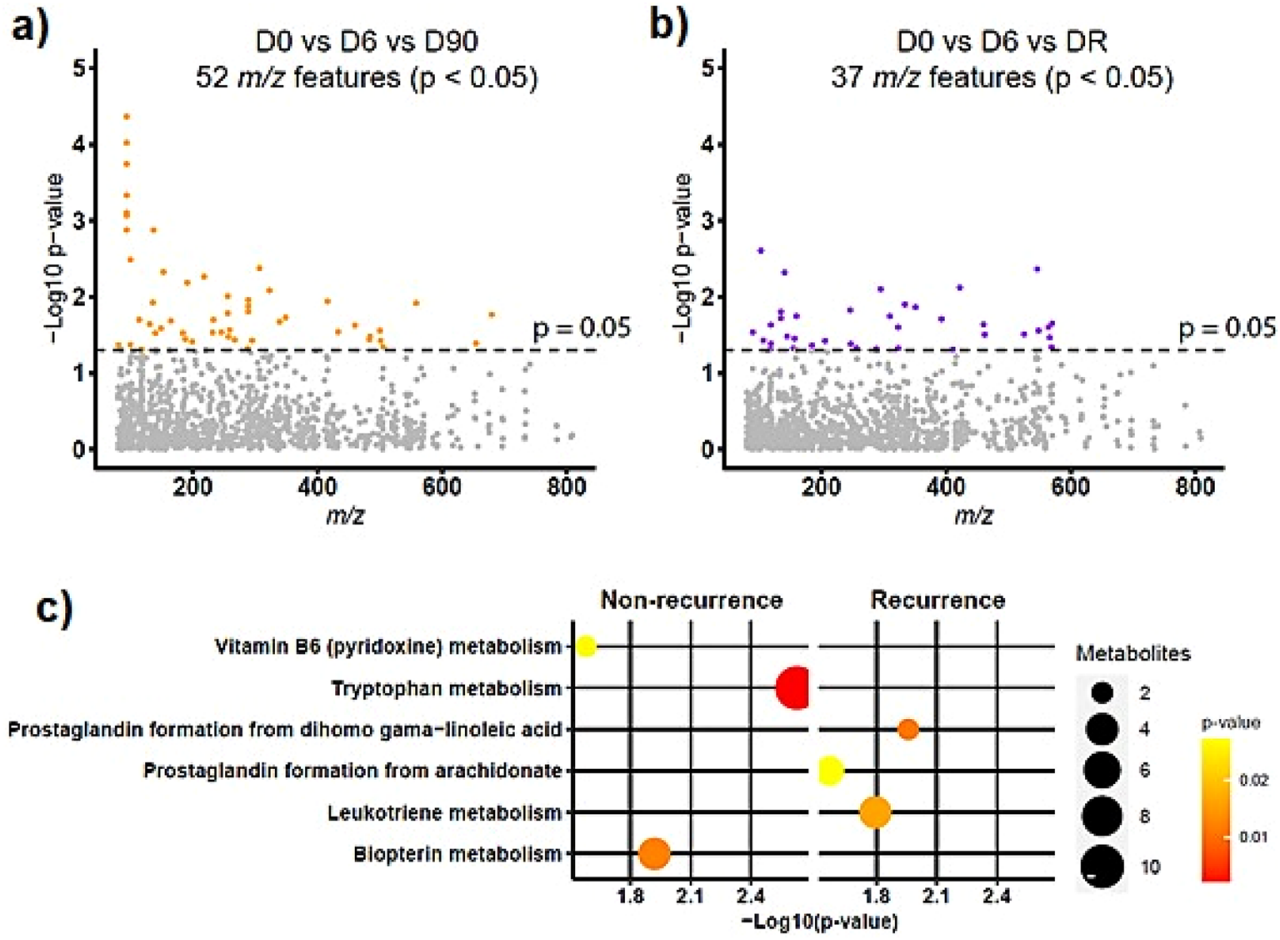
Differential abundance of metabolites features within the two groups over time. (**a**) Abundance of metabolites features over time in non-recurrent patients. (**b**) Abundance of metabolites features over time in the recurrent patients. The colored dots in both A and B graphs refer to the significant *m/z* (*p* < *0.05*) using ANOVA. (**c**) Mummichog pathway analysis of significant metabolite features in the non-recurrent and recurrent groups, including multiple adducts.

In the control group, the number of patients analyzed on different days was: D0: 57, D6: 57 and D90: 57. In the case group, they were: D0: 50, D6: 49, and DR: 49. The LC–MS analysis detected thousands of metabolite features, and after data processing and quality control analysis, a total of 1140 metabolite features were retained for downstream statistical analysis. There were 89 significant metabolite features (*p* < *0.05*), with 52 from controls and 37 from cases (Fig. 1a and b).

Mummichog analysis predicted the activity of six metabolic pathways. The control group was characterized by alterations in tryptophan, biopterin, and vitamin B6 (pyridoxine) metabolisms, whereas the case group was characterized by prostaglandin with formation from dihomo-gamma-linoleic acid, leukotriene metabolism, and prostaglandin formation from arachidonate (Fig. 1c).

**Figure 2:**
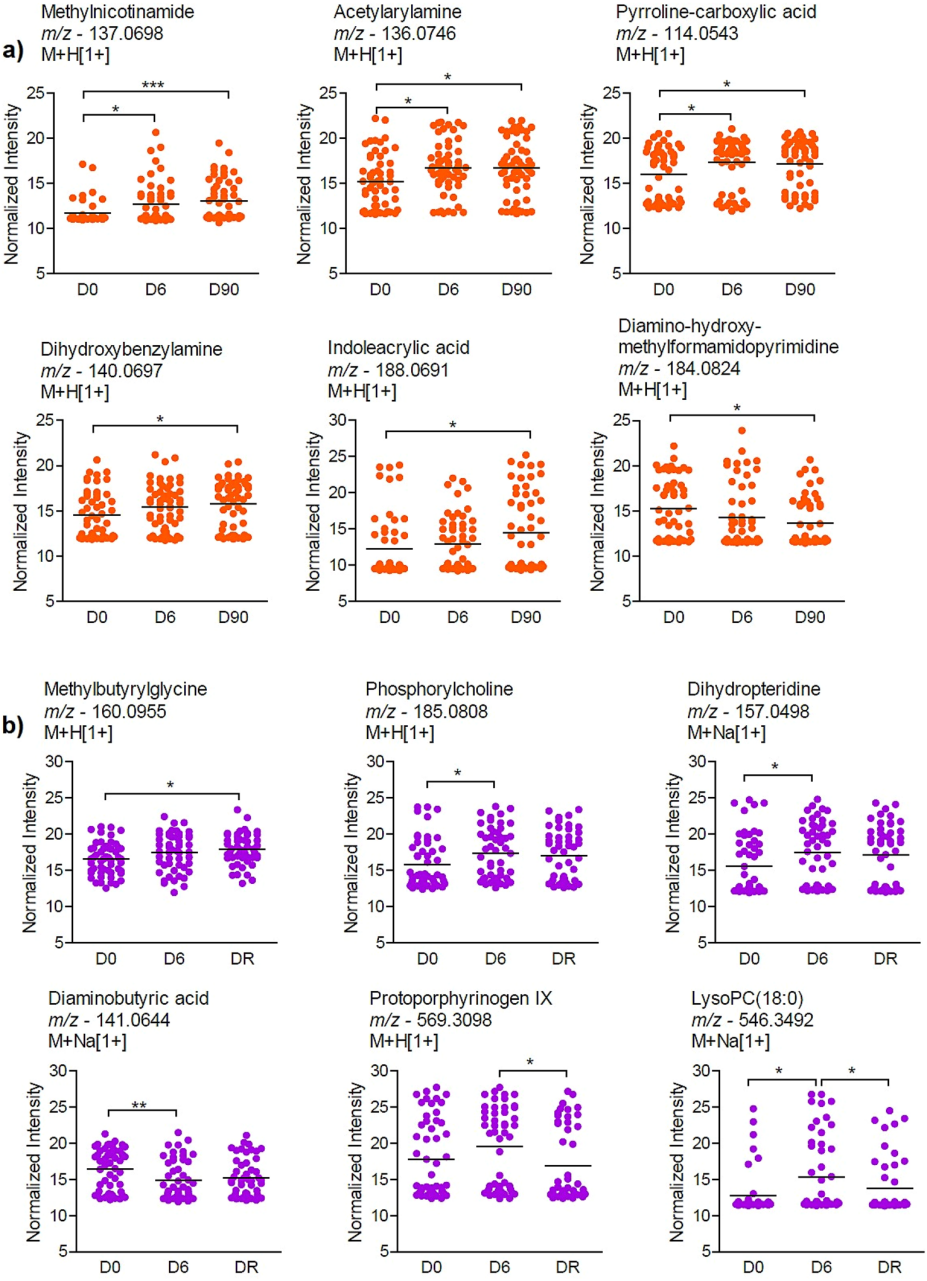
Examples of annotated metabolites over time in (**a**) the non-recurrent patients and (**b**) the recurrent patients. *p < 0.05, **p < *0.01* using ANOVA and Tukey multiple comparison test. Tentative annotations were obtained with mummichog software, including multiple adducts.

Significant metabolite features with tentative annotations are shown in Fig. 2. From the control group, the features annotated include, methylnicotinamide, acetylarylamine (benzenoid), and pyrroline-carboxylic acid (alpha amino acids) whichexhibited higher abundances with significant differences at D6 and D90 when the clinical symptoms and parasitemia had been cleared, compared to the baseline (D0) while dihydroxybenzylamine (benzenoid and human exposome) and indoleacrylic acid (indole and aromatic amino acid derivative) presented higher abundances with significant differences at D90 compared to D0 (Fig. 2a). In addition, we observed that the abundance of diamino-hydroxy-methylformamidopyrimidine was reduced with significant difference at D90 compared to D0, during the active vivax malaria, before the treatment. Almost all the metabolites annotated were either amino acids or their intermediates except methylnicotinamide which is a form of vitamin B3^31^ (Fig. 2a).

From the case group, methylbutyrylglycine displayed increased abundance with significant difference at the day of recurrence (DR) compared to D0, and the abundances of phosphorylcholine and dihydropteridine were elevated with significant differences at D6 compared to D0 (Fig. 2b). Moreover, the abundance of diaminobutyric acid reduced with significant difference at D6 compared to D0, while the abundance of protoporphyrinogen IX reduced with significant difference at DR compared to D6 (Fig. 2B). The abundance of the metabolite feature annotated as lysophosphatidylcholine (LysoPC) 18:0 was elevated with significant difference at D6 compared to both D0 and DR (Fig. 2b).

### Differential abundance of metabolite features and metabolic pathways activity between the groups

**Figure 3:**
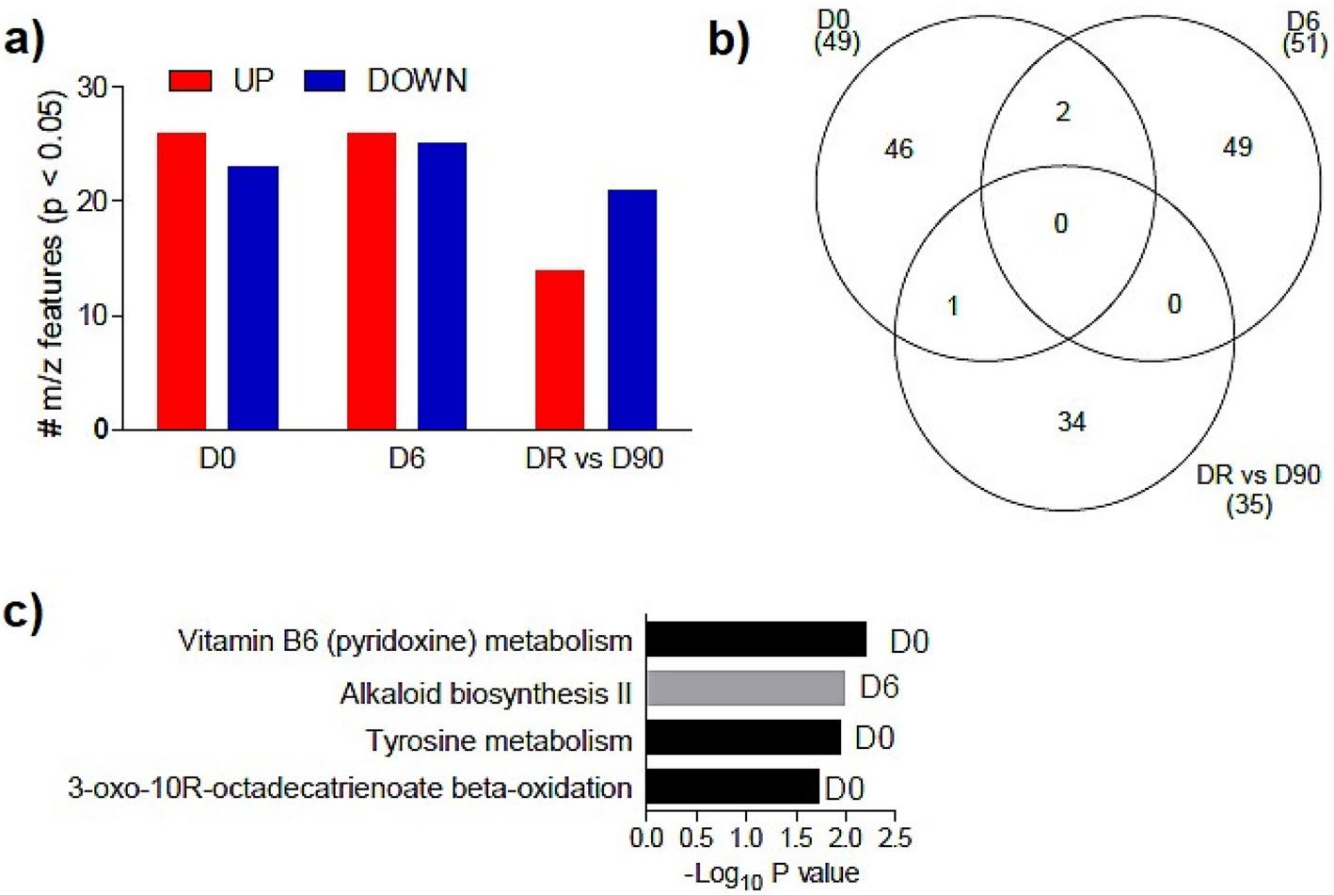
The frequency of up- and down-regulated significant metabolites over time and their metabolic pathways. (**a**) The number of significant m/z that are positively regulated and negatively regulated between groups at different times. (**b**) Venn diagram illustrating overlapping and non-overlapping differentially abundant metabolite features at different times. (**c**) Mummichog pathway analysis of pathways affected by the significant metabolite, including multiple adducts.

During active vivax malaria (D0), 26 significant metabolite features were up regulated while 23 were down regulated. At D6, when the clinical symptoms and parasitemia of most of the patients had been cleared, 26 significant metabolite features were up regulated and 25 were down regulated, whereas 13 were up regulated and 22 were down regulated at D90 (3 months after recovery) versus DR (Day of recurrence) (Fig. 3a).

In the Venn diagram, it was observed that 46, 49 and 34 metabolite features exclusively belonged to D0, D6 and D90 versus DR respectively while two metabolites features interjected between D0 and D6 and one metabolite feature interjected between D6 and DR versus D90. No metabolite was common in all the time points analyzed (Fig. 3a).

The metabolic pathways predicted by the significant metabolites in each comparison, showed that vitamin B6 (pyridoxine) metabolism, tyrosine metabolism and 3-oxo-10-octadecatrienoate β-oxidation were significant on D0, while alkaloid biosynthesis II was significant on D6. However, there were no significant pathways at the DR versus D90 (Fig. 3c).

### Differential abundance between cases and controls at the metabolite level

**Figure 4:**
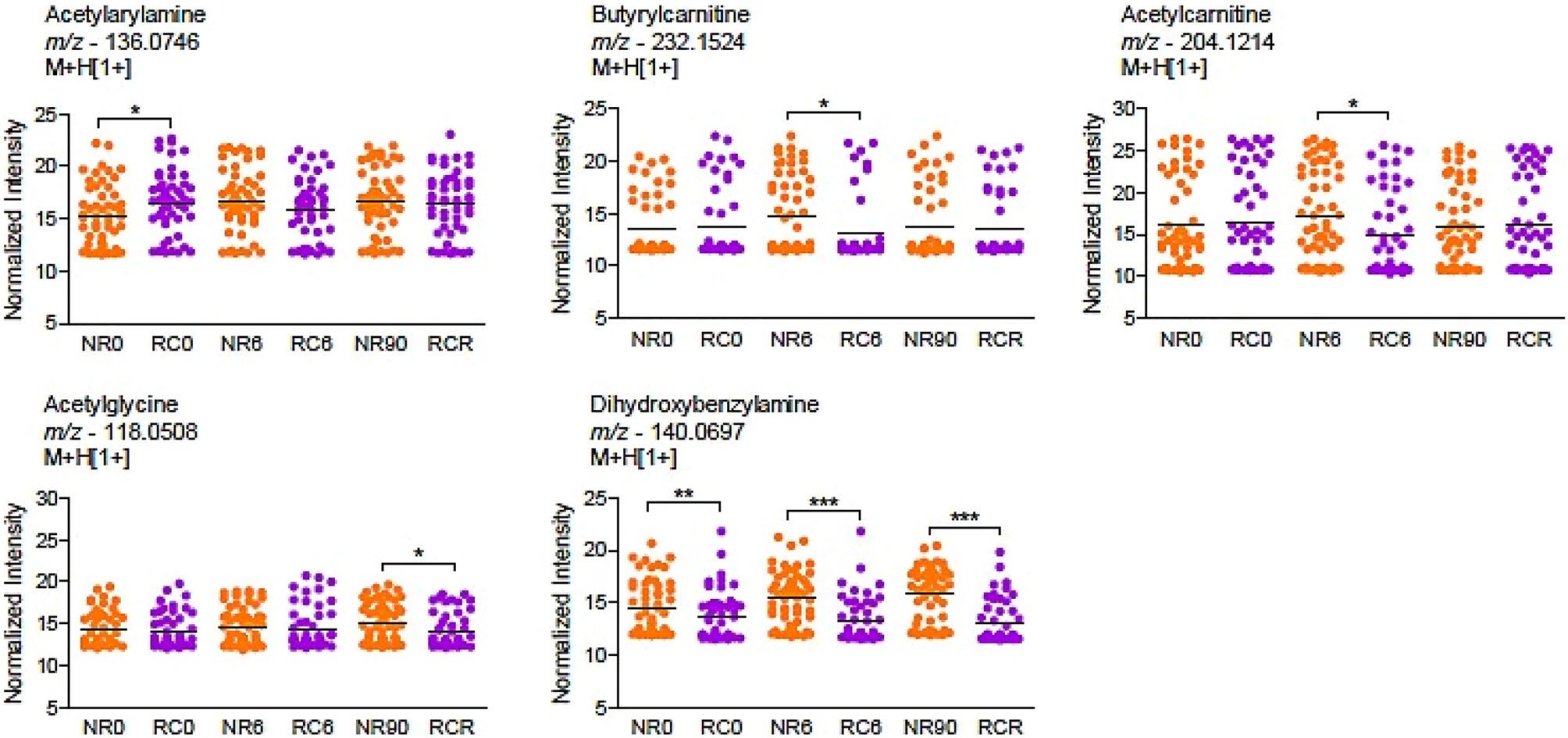
Comparisons of annotated metabolites between the two groups at the D0, D6 or DR vs D90. *p < 0.05, **p < 0.01, ***p < *0.001* using T test or Mann Whitney test (NR0: non-recurrent patients, Day0, RC0: recurrent patients, Day 0, NR6: non-recurrent patients, Day6, RC6: recurrent patients, Day6, NR90: non-recurrent patients, Day90, RCR: recurrent patients, Day of recurrence). Tentative annotations were obtained with mummichog software, including multiple adducts.

The annotated metabolites were compared between groups at different time points (D0, D6, and D90 versus DR), they include: acetylarylamine, butyrylcarnitine, acetylcarnitine, acetylglycine, and dihydroxybenzylamine (Fig. 4). Their abundances were significantly elevated in the control group compared to cases, except for acetylarylamine, whose abundance at D0 was markedly elevated in the cases. The abundance levels of butyrylcarnitine and acetylcarnitine were significantly elevated among the controls at D6. At D90, the acetylglycine levels were significantly higher in controls compared to DR. Interestingly, dihydroxybenzylamine abundance showed a significant increase in the control across all the three comparison days (D0, D6, and D90) (Fig. 4).

### Integrative analysis of metabolomics and clinical laboratory data

**Figure 5:**
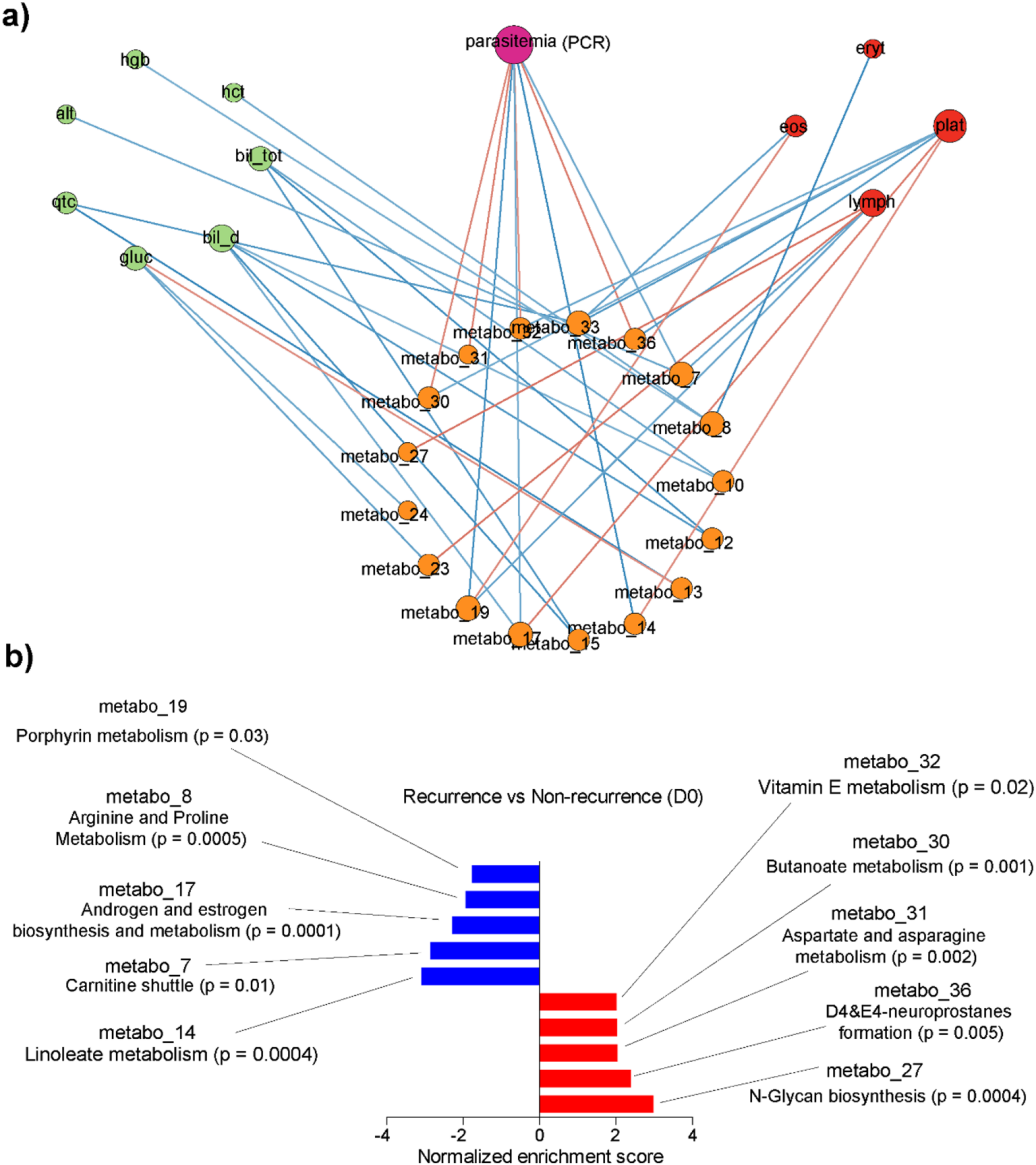
Network analysis of metabolomics and clinical laboratory data. (**a**) Integrative network composed of parasitemia, hematological, biochemical and metabolomics measurements at D0. Negative associations are depicted by blue lines and positive associations are depicted by red lines. The size of the node corresponds to the number of associations (**b**) Enrichment analysis of metabolic clusters comparing recurrence and non-recurrence at D0. Blue bars represent clusters associated with non-recurrence and red bars represent clusters associated with recurrence. Mummichog analysis was used to predict metabolic pathways enriched in clusters and only the top significant pathway is shown.

To gather another perspective into the data, we performed an integrative network analysis of hematological, parasitaemia, biochemical and metabolomics measurements collected at D0. Therefore, the dimension of metabolomics data was reduced to 36 metabolic clusters using hierarchical clustering accounting for retention time, as described^28^. Thereafter, cluster activities were used to verify associations with parasitemia, hematological and biochemical measurements with Spearman correlation. We identified a significant network composed of 29 nodes and 36 edges (p < 0.05), with parasitemia displaying the most associations with metabolic clusters, whereas biochemical measurements present mostly negative associations with metabolic clusters (Fig. 5a). We used gene set enrichment analysis to identify metabolic cluster differing between the two groups at D0. Clusters related to porphyrin metabolism (metabo_19), arginine and proline metabolism (metabo_8), androgen and estrogen biosynthesis and metabolism (metabo_17), carnitine shuttle (metabo_7) and linoleate metabolism (metabo_14) were associated with the non-recurrent phenotype, whereas recurrence was mostly related to vitamin E metabolism (metabo_32), butanoate metabolism (metabo_30), aspartate and asparagine metabolism (metabo_31), D4&E4-neuroprostanes formation and N-glycan biosynthesis (metabo_27) (Fig. 5b).

## Discussion

Metabolomics has emerged as a valuable tool in studying the relationship between hosts and parasites, enabling the discovery of information that can provide important insights for the control of malaria^]^. Through metabolomic analysis, global changes across many classes of molecules including lipids, amino acids and hemoglobin metabolites were found to impact neurologic function during cerebral malaria in *P. falciparum*^34^. Using untargeted metabolomics, we analyzed the metabolomic profile of individuals with and without malaria recurrences to understand how metabolism was affected during an active vivax malaria and recovery and at recurrence. Our findings demonstrated that malaria leads to significant changes in metabolism. We identified specific metabolites and pathways associated with recurrent participants, non-recurrent participants, or both.The metabolites identified in the non-recurrent patients include: methylnicotinamide, a metabolite of the class of nicotinamides (NAD^+^) and most species, including humans and *Plasmodium* can produce NAD^+^^35,36^. Previous studies on *Plasmodium* species reported high NAD^+^ levels during malaria, with infected erythrocytes having 5 to tenfold higher concentrations compared to the uninfected red blood cells^31,32^. In a malaria challenge study, methylnicotinamide was demonstrated to be of variable importance upon *P. falciparum* infection, being significantly higher among the Europeans than the Africans^37^. In our study, the significant elevation of methylnicotinamide at day 6 (when most patients have recovered) and day 90 (3 months after recovery) of follow up could have possibly be influenced by disruptions generated from the conversion between NAD^+^ and NADH at cellular energy production^36^. The other annotated metabolites from this group such as acetylarylamine (benzenoid), pyrroline-carboxylic acid (α-amino acids), dihydroxybenzylamine (benzenoid and human exposome), and indoleacrylic acid (indole and aromatic amino acid derivative) were largely amino acids and their intermediates. Earlier studies demonstrated that amino acids are involved in various biological processes during malaria episodes which significantly alter protein synthesis, cellular redox balance, and cause stress response^38–40^, resulting in significant decrease in their abundance^41,42^. The decrease in diamino-hydroxy-methylformamidopyrimidine from an active malaria state to becoming significantly low at full recovery suggests that the metabolite was elevated because of the disease condition. This finding is in agreement with some studies that indicated diamino-hydroxy-methylformamidopyrimidine as a potential biomarker for oxidative DNA damage^43,44^, suggesting that vivax malaria is associated with induced oxidative damage in the patients. Oxidative damage during malaria infection can arise from heme toxicity, immune response, inflammatory response and antimalarial treatment^35,45–47^. Therefore, the decrease in abundance of diamino-hydroxy-methylformamidopyrimidine following successful treatment is indicative that there was reduction in oxidative stress during the treatment and at full recovery.

In the recurrent patients, methylbutyrylglycine (acylglycine), phosphorylcholine (phospholipid) and dihydropteridine, were decreased significantly at baseline (pre-treatment) compared to either when the clinical symptoms and parasitaemia of most patients had been cleared (D6) or when the malaria had returned at day of recurrence (DR) conversely, diaminobutyric acid increased significantly at baseline compared to when the clinical symptoms and parasitemia of most patients had been cleared (D6) and when the infection had returned at day of recurrence (DR). Methylbutyrylglycine is an acylglycine, which is a metabolite of fatty acid that is used to diagnose disorders associated with mitochondrial fatty acid β-oxidation^22,23^. It can also induce oxidative lipid damage and reduce antioxidant defense in brain tissue, which has been used in diagnosing diseases related to mitochondrial fatty acids β-oxidation^24^. The impact of malaria on the enzyme responsible for acylglycine production might have led to the accumulation and increased levels of methylbutyrylglycine. According to Sengupta et al. (2011), and Kimura and Yamaguchi (1999), elevation in urinary acylglycines is an indication of impaired β-oxidation of fatty acid associated with febrile condition like malaria^22,23^, highlighting its relevance in lipid oxidative damage.

The levels of lysoPC (phospholipid) and phosphorylcholine (phospholipid related compound) decreased significantly during active infection that coincided with high parasitemia. Our observations on these metabolites reflect those by Tewari et al.(2021) in which during a malaria infection, the reduction in phosphatidylcholine metabolism allowed the parasite to sustain the synthesis of purine nucleotides by inhibiting the synthesis of phospholipids^48^. Additionally, lysoPC is said to be taken up by parasites during asexual replication and potentially trigger gametocyte production, thus providing a critical signal for increased gametocyte production and transmission^42,49,50^. Gardinassi et al. (2017) also reported that the levels of glycerophosphocholine and phosphocholine were reduced with high levels of parasitemia^5^. Similarly, this finding aligns with previous studies that have reported on the malaria parasites scavenging, modifying, and integrating fatty acids and phospholipids derived from the host^25,26^. In addition, lipids are crucial nutrients for parasite growth and for the transformation of heme to hemozoin^51^. These findings affirm the significance of lipid metabolism during malaria episodes and at the same time encourage the development of antimalarials that target the pathways involved in the uptake and metabolism of fatty acids and phospholipids^5^. In line with this and other studies, decreased levels of lysoPCs and other phospholipids predict a vivax malaria episode^5,25,26,42^. In addition, diminished levels of phosphorylcholine, dihydropteridine, hemoglobin-related protoporphyrinogen, and lysoPCs, at D0 and on DR, demonstrate how these metabolites can be indicative of *P. vivax* infection, whereas their elevated levels may suggest absence of infection^42^. The malaria infection must have generated conditions such as oxidative stress that resulted in the decrease of these metabolites^35,46^.

A comparison between groups across the follow-up days demonstrated a significant increase in acetylarylamine levels in recurrent patients and dihydroxybenzylamine levels in non-recurrent patients at baseline, revealing their potential as prognostic biomarkers for recurrent vivax malaria. Additionally, significant increases in butyrylcarnitine, acetylcarnitine, and dihydroxybenzylamine levels during post-treatment evaluations may suggest their prognostic potential for predicting recurrent malaria. There are cases of acute falciparum and vivax malaria infections in humans documented that correlate with increased levels of fatty acylcarnitines^41^. The increased levels of fatty acylcarnitines may stem from heightened utilization of the phospholipids and fatty acids in the acylcarnitine pathway during episodes of high parasitaemia. It also suggests a deficiency of the substrates essential for β-oxidation in patients with high parasitemia. Butyrylcarnitine is said to be associated with the metabolism of butyrate, a short-chain fatty acid, while acetylcarnitine is associated with the metabolism of acetyl-CoA, a key molecule in energy production and various metabolic pathways^52^. Therefore, the elevated levels of butyrylcarnitine and acetylcarnitine in plasma during malaria suggest disruptions in mitochondrial activity and fatty acid oxidation. These metabolites relating to fatty acid oxidation have been implicated as metabolites of cerebral malaria^53,54^, and rodent malaria^55^. The elevation of acetylglycine and dihydroxybenzylamine levels in patients at D90 compared to DR may be predictive of vivax malaria or absence/recovery from malaria. Acetylglycine has already been linked to malaria infection in which it has been associated with parasite induced metabolic alterations during *P. falciparum* infection^40,56^. The elevated levels of dihydroxybenzylamine at D90, compared to baseline and the day of recurrence (DR) may be predictive of presence or absence of *P. vivax* infection. Abdelrazaig et al. (2017) identified dihydroxybenzylamine in the urine of patients with malaria, as a predictive metabolite for malaria, while He et al. (1997) stated that dihydroxybenzylamine was an essential precursor for the synthesis of norepinephrine, a neurotransmitter with a significant role in physiological processes during malaria infection^57,58^.

There was no major difference in the number of up and down regulated metabolites when comparing the groups during active vivax malaria episode or during the antimalarial interventions. However, there was a large difference in the number of up and downregulated metabolites between recovered participants (3 months later) and recurrent patients (on the day of recurrence), when compared to an active malaria episode or during treatment. This observation corroborates that an individual's metabolome reflects their current health or diseased condition^16,17^.

During an active vivax malaria infection, the vitamin B6 (pyridoxine) metabolism was significantly altered. Pyridoxal 5-phosphate, the active form of vitamin B6, acts as a cofactor for several crucial enzymes found in the metabolism of *Plasmodium sp*., playing a role in protein biosynthesis, and in serine hydroxymethyltransferase (SHMT), a key enzyme in folate metabolism. This result confirmed that the vitamin B6 biosynthetic pathway is an excellent drug target^27^. Tryptophan metabolism alteration in the non-recurrent participants occurs because malaria parasite relies on the host's tryptophan as a crucial nutrient source for its survival and growth^59^. In addition, the decrease in methylnicotinamide and indolylacrylic acid abundance, which are tryptophan metabolites, as observed during the active malaria episode compared to the recovered, reaffirms this. This finding aligns with other studies that stated that tryptophan is known to decrease in the bloodstream during *Plasmodium* infections^38,60^. This observation positions tryptophan metabolism as a potential target for future antimalarial drug development^59^. Other altered metabolic pathways in the non-recurrent include prostaglandin formation from dihomo-gamma-linoleic acid and from arachidonate. Prostaglandins are a group of naturally occurring lipid compounds that play a key role in physiological processes in the human body^61^. This finding aligns with previous studies that had indicated that prostaglandins are altered by both *P. vivax* and *P. falciparum* malaria^62,63^. Biopterin metabolism was also altered in the non-recurrent participants. In the fully reduced state of biopterin (tetrahydrobiopterin (BH_4_)), it is said to be unstable during malaria due to oxidative stress, as it can non-enzymatically oxidize to dihydrobiopterin (BH_2_)^64,65^. This was clearly demonstrated in our study, where biopterin metabolism was found to be altered among the recurrent participants, and BH_2_ was one of the metabolites in the recurrent group that was significantly lower before treatment. According to Yeo et al. (2015), BH_2_ and BH_4_ oscillate between each other during malaria depending on the severity of the condition^64^. The presence of BH_2_ indicates severity because it cannot be recycled to BH_4_ due to oxidative stress. Leukotriene metabolism was one of the pathways altered in the recurrent group. Leukotrienes are formed by the 5-lipoxygenase (5-LO)-catalyzed oxidation of arachidonic acid, and they are lipid mediators that have potent proinflammatory activities^66^. This finding is in harmony with previous research that stated a decrease in abundance of leukotrienes with higher levels of parasitemia in patients infected with *P. vivax*^5^. He also stated that higher levels of parasitemia could impact the metabolism and signaling of activated leukocytes or platelets, leading to leukotriene’s perturbation^5^. Tyrosine metabolism, which is already known to play a significant role in malaria, was one of the altered metabolic pathways^41,67^. The malaria parasite depends on the host's tyrosine supply for its growth and survival^68^. Our findings confirms the importance of tyrosine metabolism to *Plasmodium* parasites and also corroborate that targeting the parasite's ability to obtain or utilize tyrosine could have antiplasmodial activity^68^. The alteration of alkaloid metabolism on day six when the patients were under medications, and most patients' parasitemia had been cleared should therefore be chloroquine related, since chloroquine is an alkaloidal drug^69^. The metabolic pathway alteration involved in the breakdown of 3-oxo-10octadecatrienoate β-oxidation aligns with the various fatty acid metabolites annotated in this study and other findings that have stated that malaria is associated with alterations in fatty acid metabolism. This reaffirms that fatty acids could be a promising target for the antimalarial drug development^5,12,22–26^.

The links between clinical and metabolic profiles during vivax malaria is not well understood. By integrating different types of data, we found significant associations between metabolic clusters that differ between recurrent patients and non-recurrent group and biochemical, cellular and parasitological parameters. In accordance to previous findings^5^, porphyrin metabolism was associated with parasitemia. Moreover, parasitemia was positively associated with metabolic clusters 30, 31, 32 and 36, all of which are significantly enriched in the recurrent phenotype. Collectively, these results suggest that features related to the parasite interaction with host account for recurrence. Among the metabolic clusters enriched for the recurrent phenotype, the most significant is related to N-glycan biosynthesis, which was also positively correlated with lymphocyte counts. Therefore, the interplay between metabolism and the immune response can be critical for the recurrence of *P. vivax* malaria.

In conclusion, this study revealed the essential metabolites in active vivax malaria episodes, recurrent vivax malaria episodes and recovered malaria patients. Additionally, altered metabolic pathways with potential therapeutic value as drug targets in the development of novel antimalarial drugs were identified. Further studies with larger cohorts representing the wider *P. vivax* geographical coverage, and comparative study of plasma metabolomics to urinary or saliva metabolomics in non-recurrent, recurrent and relapsing vivax malaria could as well advance this fight against malaria.

## Limitations

A major limitation in our study was the cohort size. However, as recurrence is an rare  phenomenon, it is difficult to have a large sample. Secondly, we were unable to determine whether the recurrence was due to relapse or reinfection. Finally, we were unable to confirm the identity of annotated metabolites, which are reported here as level 3 identification according to the Metabolomics Standard Initiative (MSI)^70^, due the unavailability of samples and the requirement for standards, reagents and dedicated equipment for new analyses. Despite these limitations, this is the first study to compare the metabolites of patients with recurrent and non-recurrent vivax malaria, and our data provide information on metabolomic differences between the two vivax malaria outcomes.

## Methods

### Study participants and sample processing

This study, while not a clinical trial, only involved participants selected from the CURAVIVAX study (ClinicalTrials.gov ID NCT03208907, first registered on 30/06/2017) an open-label, phase III, randomized to evaluate the efficacy and safety of dihydroartemisinin-piperaquine and primaquine versus chloroquine and primaquine for uncomplicated malaria by *P. vivax* monoinfection.

Individuals with *P. vivax* were admitted at the Fundação de Medicina Tropical Dr. Heitor Vieira Dourado (FMT-HVD), a reference center for infectious diseases located in Manaus, Western Brazilian Amazon. Patients were randomly assigned to one of four treatment groups: Group1- chloroquine (CQ) for 3 days + primaquine (PQ) for 14 days (0.50 mg/kg/day) concurrently, Group 2—dihydroartemisinin/piperaquine (DHA/PPQ) for 3 days + PQ for 14 days (0.50 mg/kg/day) concurrently, Group 3—CQ for 3 days + PQ for 14 days (0.50 mg/kg/day) starting on day 42 after the initial CQ, and Group 4—DHA/PPQ for 3 days + PQ for 14 days (0.50 mg/kg/day) starting on day 42 after the initial DHA/PPQ. At inclusion into the CURAVIVAX study, patient socio-demographic data including sex, age, weight, body-mass index and ethnicity was captured. Laboratory analyses were done at pre-treatment through the treatment days and follow-up including the day of recurrence.

Patients of both sexes, aged > 6 months, body weight ≥ 5 kg, with symptomatic *P. vivax* monoinfection, parasite density between 100 and 100,000 parasites/ µL were included. Exclusion criteria included the use of antimalarials in the last 60 days, mixed *Plasmodium* infections especially *P. falciparum*, pregnancy, or lactation and concomitant or underlying diseases. Patients with dengue and other febrile diseases were also excluded during screening.

After confirmation of malaria vivax by thick blood smears, whole blood and plasma samples were collected, aliquoted and stored at -80^O^C until needed for analysis. Plasma samples for day of inclusion and before treatment (D0), day six (D6), day ninety (D90) and recurrence day (DR) were used for this study.

### Sample size

For purposes of this study, convenience sampling was used to select subjects from the original study and the sample size was determined by the number of the patients with recurrence. Selected participants were grouped into case group—participants with recurrence episode during the 180-day follow-up period, and control group—individuals that did not any recurrence during this period. We selected the control group to match the case group in terms of age, sex, ethnicity, and treatment group.

### Ethics statement

This study was approved by the Fundação de Medicina Tropical-Doutor Heitor Vieira Dourado Research Ethics Committee with the certificate number: CAAE: 69476017.3.0000.0005. Informed consent was obtained from all participants. Furthermore, all methods were conducted in accordance with the relevant guidelines and regulations.

### Confirmation of *P. vivax* infection by Real-time PCR

From the whole blood samples collected on D0 and DR, genomic DNA was extracted using the Purelink-Genomic DNA Mini Kit (Qiagen, USA) according to the manufacturer`s instructions. The extracted DNA was amplified via real-time PCR using TaqMan fluorescence-labeled primers and probes to target the *P. vivax* 18S rRNA gene (Supplementary Table 1) through the Applied Biosystems 7500 Fast System as described elsewhere^71^.

### Untargeted metabolomics

Untargeted metabolomics analysis was performed as described^72,73^. Metabolites were extracted from 150 µL of sample (plasma EDTA), which was mixed with acetonitrile (2:1, v/v; − 5 °C) and centrifuged at 15,000 rpm for 15 min to remove proteins. The supernatant (90 µL) was transferred to an autosampler vial for LC–MS analysis Stable isotopes caffeine-^13^C3, tyrosine-^15^N and progesterone-d9 were used as internal standards. All analyses were performed using a High-Performance Liquid Chromatography (HPLC–UV, 1220 Infinity, Agilent Technologies) coupled with Q Exactive hybrid Quadrupole-Orbitrap mass spectrometer (Thermo Fisher). Sample extracts were injected randomly in 12 batches that included blank and quality control using a pool of the samples included in the study. All samples were analyzed with a gradient elution program and reverse phase C18 chromatography (Zorbax Eclipse Plus C18 4.6 × 150 mm 3.5 μm Agilent) with positive electrospray ionization. The binary mobile phases were water/ 0.5% formic acid with 5 mM of ammonium formate (A), and acetonitrile (B). Their gradient elution was started with 20% (B) for 5 min, then linearly increased to 100% (B) in 30 min and kept constant for 8 min in 100% (B). The eluent was restored to the initial conditions in 4 min to re-equilibrate the column, and held for the remaining 8 min. The flow rate was kept at 0.5 mL min^−1^. The injection volume for analysis was 3 μL, and the column temperature was set at 35 °C. The electrospray ionization was operating with the following settings: spray voltage 3.5 kV; capillary temperature: 269 °C; S-lens RF level 50 V; sheath gas flow rate at 53 L min^-1^; aux gas flow rate at 14 L min^-1^; sweep gas flow rate 3 L min^-1^. The mass range in the full MS scanning experiments was *m/z* 80–1200. The max IT was set at 200 ms, and AGC target was set at 1 × 10^6^. Resolving power was set at 140,000.

### Bioinformatics and statistical analyses

The raw metabolomics data was processed using *Asari* software^74^. Metabolite features were filtered by 50% presence in a determined group (recurrence or non-recurrence). Data were log2 transformed, normalized by quantile and missing values were imputed using half of the minimum intensity for each feature. The mummichog software was used to predict activity of metabolic pathways and tentative annotations of metabolites^75^. Metabolite annotation was also confirmed in METLIN Gen2 (https://metlincloud2.massconsortium.com/)^76^ using 10 ppm tolerance. According to the Metabolomics Standard Initiative (MSI), four levels of metabolite identification can be reported, including: (1) compound identification using reference chemical standards; (2) putative annotation using spectral similarity with public/commercial libraries; (3) putative annotation using accurate mass similarity with public/commercial databases; (4) unknown compounds^70^. Here, we report the putative annotation of metabolites at level 3. The integrative network analysis was performed as described previously^73^. First, metabolomics data from D0 was reduced to 36 metabolic clusters using hierarchical clustering and accounting for retention time. Following, data from parasitemia obtained via qPCR, hematological parameters and laboratory biochemical measurements were used for correlation analysis with metabolic clusters using Spearman´s method. Associations reaching a significance of p < 0.05 were used to construct the integrative network using Cytoscape v3.10 and gene set enrichment analysis was used to compare cluster activity between recurrence and non-recurrence at D0. Differential abundance was evaluated with moderated t test or moderated F test (ANOVA) using the *limma* R package. Additional statistics included Tukeys multiple-comparison test, or T test and or Mann Whitney test as appropriate.

### Supplementary Information

Supplementary Table 1.

## Data Availability

All data generated or analysed during this study are included in this published article.

## References

[1] WHO. *World Malária Report, 2022*. (2022).

[2] Popovici J (2019). Recrudescence, reinfection, or relapse? A more rigorous framework to assess chloroquine efficacy for *Plasmodium vivax* malaria. J. Infect. Dis..

[3] SIVEP-Malaria e SINAN. *O Sistema de Informação da Vigilância Epidemiológica da Malaria (SIVEP) e Sistema de Informação de Agravos de Notificação (SINAN), Brazil, 2022*. (2022).

[4] Bourgard C, Albrecht L, Kayano ACAV, Sunnerhagen P, Costa FTM (2018). Plasmodium vivax biology: Insights provided by genomics, transcriptomics and proteomics. Front. Cell. Infect. Microbiol..

[5] Gardinassi LG (2017). Metabolome-wide association study of peripheral parasitemia in Plasmodium vivax malaria. Int. J. Med. Microbiol..

[6] Lin JT (2015). Using amplicon deep sequencing to detect genetic signatures of *Plasmodium vivax* relapse. J. Infect. Dis..

[7] Wells TNC, Burrows JN, Baird JK (2010). Targeting the hypnozoite reservoir of Plasmodium vivax: The hidden obstacle to malaria elimination. Trends Parasitol..

[8] Mueller I (2009). Key gaps in the knowledge of Plasmodium vivax, a neglected human malaria parasite. Lancet Infect. Dis..

[9] Battle KE (2014). Geographical variation in Plasmodium vivax relapse. Malar. J..

[10] Zuluaga-Idárraga L (2016). Prospective study of Plasmodium vivax malaria recurrence after radical treatment with a chloroquine-primaquine standard regimen in Turbo, Colombia. Antimicrob. Agents Chemother..

[11] Melo GC (2014). Expression levels of pvcrt-o and pvmdr-1 are associated with chloroquine resistance and severe Plasmodium vivax malaria in patients of the Brazilian Amazon. PLoS ONE.

[12] Uppal K (2017). Plasma metabolomics reveals membrane lipids, aspartate/asparagine and nucleotide metabolism pathway differences associated with chloroquine resistance in Plasmodium vivax malaria. PLoS ONE.

[13] WHO. World malaria report 2009. (2009).

[14] Tasman H (2022). Assessing the impact of relapse, reinfection and recrudescence on malaria eradication policy: A bifurcation and optimal control analysis. Trop. Med. Infect. Dis..

[15] Adekunle AI (2015). Modeling the dynamics of Plasmodium vivax infection and hypnozoite reactivation in vivo. PLoS Negl. Trop. Dis..

[16] Tan S-L, Ganji G, Paeper B, Proll S, Katze MG (2007). Systems biology and the host response to viral infection. Nat. Biotechnol..

[17] Lee Y (2015). Systems biology from virus to humans. J. Anal. Sci. Technol..

[18] Rahman M, Hasan MR (2014). Pentose phosphate pathway in disease and therapy. Adv. Mater. Res..

[19] Lucchi NW, Oberstaller J, Kissinger JC, Udhayakumar V (2013). Malaria diagnostics and surveillance in the post-genomic era. Public Health Genom..

[20] Beri D (2019). Insights into physiological roles of unique metabolites released from Plasmodium-infected RBCs and their potential as clinical biomarkers for malaria. Sci. Rep..

[21] Zhou M, Varol A, Efferth T (2021). Multi-omics approaches to improve malaria therapy. Pharmacol. Res..

[22] Sengupta A (2011). Global host metabolic response to Plasmodium vivax infection: A 1H NMR based urinary metabonomic study. Malar. J..

[23] Kimura M, Yamaguchi S (1999). Screening for fatty acid beta oxidation disorders Acylglycine analysis by electron impact ionization gas chromatography–mass spectrometry. J. Chromatogr. B.

[24] Knebel LA (2012). Methylbutyrylglycine induces lipid oxidative damage and decreases the antioxidant defenses in rat brain. Brain Res..

[25] Moll GN (1988). Phospholipid uptake by *Plasmodium knowlesi* infected erythrocytes. FEBS Lett..

[26] Krishnegowda G, Gowda DC (2003). Intraerythrocytic Plasmodium falciparum incorporates extraneous fatty acids to its lipids without any structural modification. Mol. Biochem. Parasitol..

[27] Kronenberger T (2014). Vitamin B6-dependent enzymes in the human malaria parasite *Plasmodium falciparum*: A druggable target?. BioMed Res. Int..

[28] Gardinassi LG (2018). Integrative metabolomics and transcriptomics signatures of clinical tolerance to Plasmodium vivax reveal activation of innate cell immunity and T cell signaling. Redox Biol..

[29] Jain P, Chakma B, Patra S, Goswami P (2014). Potential biomarkers and their applications for rapid and reliable detection of malaria. BioMed Res. Int..

[30] Kaur H (2018). Screening and identification of potential novel biomarker for diagnosis of complicated Plasmodium vivax malaria. J Transl Med.

[31] Cheda A (2021). A derivative of vitamin B3 applied several days after exposure reduces lethality of severely irradiated mice. Sci. Rep..

[32] Yu X, Feng G, Zhang Q, Cao J (2021). From metabolite to metabolome: Metabolomics applications in plasmodium research. Front. Microbiol..

[33] Salinas JL, Kissinger JC, Jones DP, Galinski MR (2014). Metabolomics in the fight against malaria. Mem. Inst. Oswaldo Cruz.

[34] Gupta S (2017). Extensive alterations of blood metabolites in pediatric cerebral malaria. PLoS ONE.

[35] Vasquez M, Zuniga M, Rodriguez A (2021). Oxidative stress and pathogenesis in malaria. Front. Cell. Infect. Microbiol..

[36] O’Hara JK (2014). Targeting NAD+ metabolism in the human malaria parasite plasmodium falciparum. PLoS ONE.

[37] Betouke Ongwe ME (2022). Urinary metabolic profiling in volunteers undergoing malaria challenge in gabon. Metabolites.

[38] Leopold SJ (2019). Amino acid derangements in adults with severe falciparum malaria. Sci. Rep..

[39] Sengupta A, Basant A, Ghosh S, Sharma S, Sonawat HM (2011). Liver metabolic alterations and changes in host intercompartmental metabolic correlation during progression of malaria. J. Parasitol. Res..

[40] Tewari SG, Swift RP, Reifman J, Prigge ST, Wallqvist A (2020). Metabolic alterations in the erythrocyte during blood-stage development of the malaria parasite. Malar. J..

[41] Colvin HN, Joice Cordy R (2020). Insights into malaria pathogenesis gained from host metabolomics. PLoS Pathog..

[42] Cordy RJ (2019). Distinct amino acid and lipid perturbations characterize acute versus chronic malaria. JCI Insight.

[43] Greenberg MM (2012). The formamidopyrimidines: Purine lesions formed in competition with 8-oxopurines from oxidative stress. Acc. Chem. Res..

[44] Jiranusornkul S, Laughton CA (2008). Destabilization of DNA duplexes by oxidative damage at guanine: Implications for lesion recognition and repair. J. R. Soc. Interface.

[45] Percário S (2012). Oxidative stress in malaria. Int. J. Mol. Sci..

[46] Thomas DC (2017). The phagocyte respiratory burst: Historical perspectives and recent advances. Immunol. Lett..

[47] Lingappan K (2018). NF-κB in oxidative stress. Curr. Opin. Toxicol..

[48] Tewari SG (2021). Metabolic survival adaptations of plasmodium falciparum exposed to sublethal doses of fosmidomycin. Antimicrob. Agents Chemother..

[49] Brancucci NMB (2017). Lysophosphatidylcholine regulates sexual stage differentiation in the human malaria parasite Plasmodium falciparum. Cell.

[50] Ramaprasad A (2022). A choline-releasing glycerophosphodiesterase essential for phosphatidylcholine biosynthesis and blood stage development in the malaria parasite. eLife.

[51] Ambele MA, Egan TJ (2012). Neutral lipids associated with haemozoin mediate efficient and rapid β-haematin formation at physiological pH, temperature and ionic composition. Malar. J..

[52] Cao Y (2009). Comparison of pharmacokinetics of L-carnitine, Acetyl-L-carnitine and Propionyl-Lcarnitine after single oral administration of L-carnitine in healthy volunteers. Clin. Investig. Med..

[53] Gordon EB (2015). Targeting glutamine metabolism rescues mice from late-stage cerebral malaria. Proc. Natl. Acad. Sci. U. S. A..

[54] Pappa V (2015). Lipid metabolites of the phospholipase A2 pathway and inflammatory cytokines are associated with brain volume in paediatric cerebral malaria. Malar. J..

[55] Basant A, Rege M, Sharma S, Sonawat HM (2010). RAesletaercrhations in urine, serum and brain metabolomic profiles exhibit sexual dimorphism during malaria disease progression. Malar. J..

[56] Andrade CM (2020). Increased circulation time of Plasmodium falciparum underlies persistent asymptomatic infection in the dry season. Nat. Med..

[57] Abdelrazig S (2017). A metabolomic analytical approach permits identification of urinary biomarkers for Plasmodium falciparum infection: A case–control study. Malar. J..

[58] He H, Stein CM, Christman B, Wood AJJ (1997). Determination of catecholamines in sheep plasma by high-performance liquid chromatography with electrochemical detection: Comparison of deoxyepinephrine and 3,4-dihydroxybenzylamine as internal standard. J. Chromatogr. B Biomed. Sci. Appl..

[59] Modoux M, Rolhion N, Mani S, Sokol H (2021). Tryptophan metabolism as a pharmacological target. Trends Pharmacol. Sci..

[60] Farinella DN (2023). Malaria disrupts the rhesus macaque gut microbiome. Front. Cell. Infect. Microbiol..

[61] Ricciotti E, FitzGerald GA (2011). Prostaglandins and inflammation. Alter. Thromb. Vasc. Boil.

[62] Andrade BB (2010). Heme impairs prostaglandin E2 and TGF-β production by human mononuclear cells via Cu/Zn superoxide dismutase: Insight into the pathogenesis of severe malaria. J. Immunol..

[63] Keller CC (2006). Suppression of prostaglandin E2 by malaria parasite products and antipyretics promotes overproduction of tumor necrosis factor–a: Association with the pathogenesis of childhood malarial anemia. J. Infect. Dis..

[64] Yeo TW (2015). Impaired systemic tetrahydrobiopterin bioavailability and increased dihydrobiopterin in adult falciparum malaria: Association with disease severity, impaired microvascular function and increased endothelial activation. PLoS Pathog..

[65] Rubach MP (2015). Impaired systemic tetrahydrobiopterin bioavailability and increased oxidized biopterins in pediatric falciparum malaria: Association with disease severity. PLoS Pathog..

[66] Rogerio AP, Anibal FF (2012). Role of leukotrienes on protozoan and Helminth infections. Mediat. Inflamm..

[67] Xie SC (2022). Reaction hijacking of tyrosine tRNA synthetase as a new whole-of-life-cycle antimalarial strategy. Science.

[68] Kesely K (2020). Identification of tyrosine kinase inhibitors that halt Plasmodium falciparum parasitemia. PLoS ONE.

[69] Uzor PF (2020). Alkaloids from plants with antimalarial activity: A review of recent studies. Evid.-Based Complement. Altern. Med..

[70] Sumner LW (2007). Proposed minimum reporting standards for chemical analysis: Chemical Analysis Working Group (CAWG) Metabolomics Standards Initiative (MSI). Metabolomics.

[71] Almeida ACG (2018). High proportions of asymptomatic and submicroscopic Plasmodium vivax infections in a peri-urban area of low transmission in the Brazilian Amazon. Parasites Vectors.

[72] Gardinassi LG (2023). Integrated metabolic and inflammatory signatures associated with severity of, fatality of, and recovery from COVID-19. Microbiol. Spectr..

[73] Mwangi VI (2023). Methylprednisolone therapy induces differential metabolic trajectories in severe COVID-19 patients. mSystems.

[74] Li S, Siddiqa A, Thapa M, Chi Y, Zheng S (2023). Trackable and scalable LC-MS metabolomics data processing using asari. Nat. Commun..

[75] Li S (2013). Predicting network activity from high throughput metabolomics. PLoS Comput. Biol..

[76] Domingo-Almenara X (2019). The METLIN small molecule dataset for machine learning-based retention time prediction. Nat. Commun..

