# Supplementary material for: Host metabolomic responses in recurrent P. vivax malaria
Source: Sci Rep. 2024 Mar 27;14:7249. doi: 10.1038/s41598-024-54231-5 (PMC10973444; doi:10.1038/s41598-024-54231-5)
Supplement: Supplementary file 1 — Supplementary Table 1. [file 41598_2024_54231_MOESM1_ESM.docx]

| **Table S1. Oligonucleotide primers and probes used in qPCR assays.** | | |
| --- | --- | --- |
| **Target gene** | **Primers/ Probes** | **Sequence 5’- 3’** |
| Pv 18S rRNA | Vivax_Fw | GCT TTG TAA TTG GAA TGA TGG GAA T |
|  | Vivax_Rev | ATG CGC ACA AAG TCG ATA CGA AG |
|  | Vivax_Probe | **VIC** – AGC AAC GCT TCT AGC TTA – **MGB** – **NFQ** |
| Almeida *et al*., 2018 | | |
